# Evaluation of the Psychometric Properties of the Movement Assessment Battery Second Edition (M-ABC2): A Cross-Sectional Study

**DOI:** 10.3390/children11050555

**Published:** 2024-05-05

**Authors:** Eleonora Scarfò, Michela Ranucci, Anna Berardi, Rachele Simeon, Giovanni Galeoto

**Affiliations:** 1 Department of Human Neurosciences, Sapienza University of Rome, 00185 Rome, Italy; scarfo.1795896@studenti.uniroma1.it (E.S.); ranucci.1752024@studenti.uniroma1.it (M.R.); anna.berardi@uniroma1.it (A.B.); rachele.simeon@uniroma1.it (R.S.); 2 IRCSS Neuromed, Via Atinense, 18, 86077 Pozzilli, Italy

**Keywords:** school, participation, environment, disability, assessment, cultural adaptation, validation

## Abstract

This cross-sectional study assessed the psychometric properties of the movement assessment battery for children second edition (M-ABC2) in Italian children with typical development, focusing on reliability and percentile rank establishment. The M-ABC2 is widely utilized but lacks validation in Italian populations. One hundred and eight children were recruited. Test-retest reliability was evaluated using intra-class correlation coefficients (ICCs), indicating good to excellent reliability across age groups, albeit with outliers requiring further scrutiny. Standard scores and percentile ranks were established, revealing age-related variations in motor performance. Noteworthy differences in completion times and error rates were observed among the age groups, highlighting the dynamic nature of motor development. While the findings provide valuable insights for clinicians and researchers, limitations such as sample size and geographic representation should be addressed in future studies. This study underscores the importance of employing reliable assessment tools for comprehensive understanding and effective intervention in pediatric motor development.

## 1. Introduction

Movement skills are essential for children’s daily activities. They are fundamental, for interacting with friends or participating in school activities [1].

During the early stages of development, motor skills are necessary for exploring the surrounding environment, while later in life, both gross and fine motor skills are crucial for performing various tasks, including self-help skills like dressing or writing [2].

Children with motor impairments may have challenges performing activities of daily living (ADL), such as academic, social, and house-related activities. Low self-esteem, low self-confidence, anxiety, and social isolation are some of the problems that children with atypical motor development may experience [3].

Different tools to assess movement performance in childhood and adolescence are available [4,5,6].

To discriminate the motor performance of typical and atypical children, choosing the appropriate test that evaluates motor skills adequately and fairly is necessary based on its validity and reliability. Validated measurement tools are essential in the assessment stage to learn about the child’s initial status, build an evidence-based diagnosis, determine the goals and treatment plan, and, finally, measure treatment effectiveness. Additionally, the use of standardized and validated instruments enables the assessment of the level of development of the population with neurodevelopmental disorders compared to that of the general population. Validating a tool on a healthy population before its use on an affected population is crucial for several reasons. Firstly, it ensures the stability and reliability of the scale by assessing its effectiveness in measuring the intended abilities or characteristics among individuals without health conditions [7]. Additionally, data collected from healthy subjects can establish reference values, aiding in interpreting the results from the affected population [8]. Evaluating the acceptability and practicality of the assessment tool on healthy individuals helps determine its usability in clinical or research settings [9]. Lastly, validating the scale on a healthy population before its use on an affected population minimizes the risk of bias, ensuring that the results are accurate and reliable [10]. This ensures that assessment tools are valid and reliable before making clinical or diagnostic decisions.

The movement assessment battery for children (M-ABC) is one of the most widely studied and validated assessment scales in several languages [11,12,13,14,15,16]. The M-ABC2 is a battery that comprehensively measures motor skills, which was developed based on a normative sample in the UK by Henderson et al. as a revision of the previous version [17]. It has undergone numerous validation studies in various populations and cultural contexts. The M-ABC2 has been translated into many languages and is widely used in clinical and research studies worldwide, making it one of the most reliable and valid scales for assessing motor skills in children. The M-ABC2 has been studied in various populations, including children without motor issues. For instance, its validity and reliability have been investigated in Iranian children [12]. The tool was also assessed in a cohort of children in the Netherlands, indicating that the updated assessment can effectively evaluate motor performance in typically developing three-year-olds [18]. The study by Jaikaew et al. established typical scores achieved by German children on the M-ABC to provide a basis for comparison for professionals in interpreting results and making motor diagnoses in German children [14]. A recent study also analyzed the applicability of the tool in children aged 3–6 years in Taiwan, comparing the results with those of a standardized sample population in the United Kingdom [11]. 

Validation and cultural adaptation have shown that this tool is valid and reliable in all languages it has been translated into; however, item differences depend on the country. For example, the test administration time differed by ten seconds for each item between Chinese and Greek children. Test-retest reliability was performed in both typical children [19,20]. The results indicated a difference of 1 standard deviation despite the children’s ability to complete the tests. It is, therefore, critical to verify performance with normative data [21].

These studies demonstrate the usefulness of the M-ABC in assessing motor skills in children with typical development and providing valuable baseline data for interpreting scale results in different populations. 

Currently, the M-ABC2 is available in Italian, but there are no validation studies of the psychometric properties in children with typical development. 

For this reason, this cross-sectional study aimed to assess the psychometric properties of the M-ABC2 in a population of Italian children with typical development, measuring the test-retest reliability and establishing percentile ranks for the different subscales. Reference percentiles were calculated for the different subscales of the M-ABC to provide a normative context for evaluating children’s motor performance. Using the data collected from the reference population, reference scores corresponding to specific percentiles were determined, enabling a comparative assessment of children’s motor skills based on their age.

## 2. Materials and Methods

### 2.1. Participants

The participants were enrolled at the Human Neurosciences department of Sapienza Università di Roma from May 2023 to October 2023. The inclusion criteria were ages between 3 and 16 years, the absence of known medical conditions or disorders, and written informed consent obtained from the children’s parents. All children were assessed individually according to the test rules. The parents or guardians provided written informed consent, indicating that the children participated voluntarily and could withdraw from participation at any time without providing a reason. It also stated that anonymity was guaranteed and that the data would be protected. The participants were treated according to the principles outlined in the Helsinki Declaration, which ensured ethical conduct throughout the study.

In the literature, sample size recommendations range from 2 to 20 subjects per item [22]. In the articles analyzed in a systematic review of sample size used to validate a scale, the mean subject-to-item ratio was 28, with a minimum of 1 and a maximum of 527 [22]. Furthermore, Shoukri et al. [23] reported, “However, in many cases, values of the reliability coefficient under the null and alternative hypotheses may be difficult to specify. Under such circumstances, one can safely recommend only two or three replications per subject”. Consistent with previous studies of the M-ABC2 and according to the recommendations available in the literature, the authors of this study considered a minimum sample size of 30 subjects adequate. The sample estimation was decided considering the validations of the M-ABC 2 in the other languages [11,12,14,16,19,20,21].

### 2.2. Movement Assessment Battery Second Edition (M-ABC2)

The movement ABC test is designed to be administered individually within a setting with specific characteristics [17]. 

The assessment room should measure at least 6 m × 4 m and have a smooth, white wall. Part of the floor surface should be relatively hard and smooth, and the space should also be equipped with at least one table and two chairs to adequately perform manual dexterity tasks. 

It is recommended that appropriate physical education clothing be used so that the movements are not impaired and are easily observed.

The test aims to classify children between the ages of 3 and 16 years and 11 months according to the degree of motor impairment; when performing the movement ABC-2 battery test, the child or young person is asked to perform a series of eight standardized motor tasks. The battery consists of three subtests, each of which consists of several items that measure three different areas:

- Manual dexterity (three items);
- Aiming and grasping (two items);
- Balance (three items).

The item scores should be transformed into standard scores (mean = 10). Some tasks must be performed first with the preferred hand/leg and then with the non-preferred one. The score for these tasks is calculated by averaging the two attempts.

The total test score (TTS) is calculated by summing the standard scores of the eight items (range = 8–152). Standard scores are provided in the TTS manual (mean = 10; SD = 3) both for the three sections of the test and the total score, adjusted for age and percentiles and broken down by year of age from 5 to 16 years and by semester of age from 3 to 4.11 years. 

It also provides quantitative and qualitative information on how the child or young person copes with and performs these tasks and, thus, on movement skills. 

The test is divided into 3 age groups:

- Age band 1: 3 to 6 years old;
- Age band 2: 7 to 10 years old;
- Age band 3: 11 to 16 years old.

The administration time of the eight tasks varies from 20 to 40 min, depending on the subject’s age, the degree of difficulty, and the examiner’s experience.

### 2.3. Procedures

The initial administration, conducted by two independent operators who were not authors, played a crucial role in confirming the scale’s independence from raters and the non-influence of the administration differences on the obtained scores.

The scale’s reliability and validity were evaluated using the “Consensus-Based Standards for the Selection of Health Status Measurement Instruments” (COSMIN) [24] checklist. 

The test-retest reliability was evaluated by measuring the stability of the single items when carried out at different times (test-retest), at the end of which the intra-class correlation coefficient (ICC) was calculated. A time interval of 48 h was considered appropriate for the current population in accordance with previous validation and cultural adaptation studies of the same test. Based on the 95% confidence interval of the ICC estimate, values less than 0.5, between 0.5 and 0.75, between 0.75 and 0.9, and greater than 0.90 were indicative of poor, moderate, good, and excellent reliability, respectively [25,26]. 

Standard scores are used to assess an individual’s relative position within a reference distribution; they are calculated so that the mean is 100 and the standard deviation is typically 15 or 16, allowing for a uniform comparison of the results across individuals or groups. Percentiles are a statistical concept used to describe the position of a particular value within a dataset relative to the rest of the values. They divide a distribution into one hundred equal parts. Percentiles help us to understand how an individual’s performance compares to others in the same population.

All statistical analyses were performed using IBM SPSS Statistics for Windows (version 23.0; IBM Corp., Armonk, NY, USA).

## 3. Results

As reported in Table 1, a cohort of 108 participants, comprising 69 males, was enlisted. The sample under scrutiny mirrored the Italian populace concerning key sociodemographic traits such as ethnicity and parental education levels. However, it is crucial to acknowledge that the geographical dispersion might need full representation, considering the confinement of recruitment to two territorial services within the Rome region.

The test-retest reliability results are delineated for each age group, facilitating a nuanced examination of the measurement consistency across different age spans. As indicated in Table 2, the intra-rater test-retest reliability exhibited a good level, with an interclass coefficient (ICC) ranging from 0.823 to 0.975 within the 3 to 6.11 age group, and similarly within the 7 to 10.11 age group (ICC range: 0.839 to 0.940), except for the outliers identified in items 3 (ICC = 0.111) and 6 (ICC = 0.039). In the third age band, correlation coefficients varied from moderate to good (0.517 to 0.887), excluding items 3 and 8, which yielded correlations of 0.233 and 0.140, respectively. 

Table 3 illustrates the inter-rater test-retest reliability across each band, demonstrating excellent reliability. In band 1, the ICC ranges from 0.964 to 1, while in band 2, it varies from 0.902 to 1, and in band 3, it ranges from 0.943 to 1.

Table 4, Table 5 and Table 6 show the standard scores for each age group.

Table 4 presents the results for the first age group (3–6.11 years). The maximum time to complete the first task was 30 s for the preferred hand and 32 s for the non-preferred hand. Regarding the second item (bimanual manual dexterity activity), the maximum completion time for this age group was 150 s; in comparison, the minimum time was 16.9 s, indicating that almost half of the population completed the task in less than a minute. Regarding the third item on manual dexterity, half of the population made fewer than five errors, with a maximum of seventeen errors recorded. As for the “Aiming and Grasping” subscale, the minimum score was two, as 10% of children scored zero on both items in the domain. Finally, concerning the last subscale, “Balance,” the scores for item 6 indicate no difference between the preferred and non-preferred leg, while item 8 highlights that 40% of the population achieved the maximum score of five.

Table 5 presents the standard scores for the second age group (7–10.11), which includes 17 children. In contrast to “Band 1”, the first item of the “Manual dexterity” subscale demonstrates, for the minimum score of one, a noticeable difference in terms of time between the preferred and non-preferred hand. A child aged between 7 and 10.11 years achieving a time of 50 s with the preferred hand received a score of one, whereas achieving the same time with the non-preferred hand yielded a score of eight. Similarly, 45% of children made fewer than five errors within the second age group. Regarding the “Aiming and Grasping” subscale, akin to the first age group, the minimum attributable score was two, as only 10% of the population scored zero. The last subscale underscores that, for the final item, a score lower than two could not be assigned, as no child scored 0.

Table 6 presents the results of the third age group, comprising 20 children aged between 11 and 16.11 years. In this instance, no significant difference in timings between the preferred and non-preferred hands is observed. Concerning the third item of manual dexterity, it is noteworthy that 11 out of 20 of the reference population committed zero errors while performing task number three. Regarding the “Aiming and Grasping” subscale, the minimum attributable score for item 4 executed with the preferred hand was four, as the minimum score obtained was five, achieved by 4 out of 20 of the population. Conversely, the minimum attributable score for the non-preferred hand was one, as 10% of the population obtained a score of three. The “Balance” subscale shows that three-quarters of the population scored five in performing item 8 with the preferred leg, equating to a standard score of nineteen; this score differs by 10% when performed with the non-preferred leg.

Table 7 shows the scores grouped by percentiles.

## 4. Discussion

This cross-sectional study aimed to assess the psychometric properties of the M-ABC2 in a population of Italian children with typical development, measuring test-retest reliability and establishing percentile ranks for the different subscales.

The study demonstrates promising test-retest reliability across various age groups, as indicated by the inter-class correlation coefficients (ICCs) ranging from 0.823 to 0.975. These findings suggest consistent measurement outcomes over time, reinforcing the reliability of the assessment tool. However, the presence of outliers in specific items necessitates further investigation into potential sources of variability and the validity of these measures. Notably, outliers in the ICCs, indicative of potential variability in test-retest reliability, were predominantly observed within age groups two and three. This phenomenon may stem from the limited sample size or the possibility of healthy children exhibiting immediate performance enhancements upon repeating the same task, a phenomenon commonly observed in psychometric assessments [27,28]. Previous research has also suggested that individual differences in motor development and learning capabilities may contribute to variations in test-retest reliability across different age groups [29]. However, further investigation is warranted to fully elucidate the underlying factors influencing the presence of outliers in correlation coefficients within specific age cohorts. 

While the M-ABC2 is globally used, some psychometric issues have been identified in various countries. For example, in Germany, Wagner et al. [30] found problematic models in four motor tasks, raising concerns about discriminant and convergent validities. Similarly, in China, authors concluded that the reproducibility and validity of age band 1 in the M-ABC2 were poor, suggesting the need for adjustments to improve the test’s psychometric properties [31]. In Brazil, an analysis of M-ABC2 multidimensionality for children aged 7–10 years indicated the necessity of excluding three subtests to achieve a better-adjusted model [15]. However, there is a gap in the literature, particularly in Brazil, regarding a detailed adequacy analysis of the M-ABC2 for children aged 3–5 years. This gap was emphasized by Brown [16], who highlighted issues regarding context, item transition, and the assessment of one age group at a time.

The analysis reveals age-related differences in performance across various tasks, emphasizing the importance of considering developmental trajectories in assessing motor skills and dexterity. Differences in completion times and error rates were observed across different age bands, underscoring the dynamic nature of motor development during childhood and adolescence. The interpretation of the outcomes must consider the study limitations related to the small sample size and inadequate sample stratification within each age group. The findings suggest the need for further refinement of assessment tools to accommodate a broader range of skill levels.

Future research endeavors could explore additional psychometric properties of the M-ABC2, such as internal consistency and concurrent validity. Longitudinal studies tracking motor development across multiple age points could provide valuable insights into the trajectory of motor skill acquisition and its relationship with other developmental domains. Additionally, efforts should be made to recruit larger and more diverse samples to enhance the generalizability of the findings and address potential psychometric limitations.

## 5. Conclusions

The standardized scores offer invaluable insights for clinicians and researchers engaged in pediatric assessments. Professionals can effectively pinpoint developmental delays or abnormalities by comprehending normative performance levels within specific age ranges, tailoring intervention strategies, and monitoring progress over time. Moreover, identifying the ceiling effects observed in certain items underscores the necessity for further refinement of assessment tools to accommodate a broader range of skill levels. While the sample characteristics align with key sociodemographic traits of the Italian population, the limited geographical distribution may pose challenges to the generalizability of the findings. Future studies should prioritize recruiting participants from diverse geographical regions to ensure broader representativeness and enhance the external validity of the results.

Expanding upon the current findings, future research endeavors could delve into additional psychometric properties of the assessment tool, such as internal consistency and concurrent validity. Additionally, longitudinal studies tracking motor development across multiple age points could yield valuable insights into the trajectory of motor skill acquisition and its interplay with other developmental domains.

In summary, the findings presented in this study significantly contribute to our understanding of motor development and emphasize the critical importance of employing reliable and valid assessment tools in both pediatric practice and research settings.

## Figures and Tables

**Table 1:** Demographic characteristics of the included participants.

Population (n = 108)	Frequency (%)
**Age range**	
3–6.11	71 (65.7)
7–10.11	17 (15.7)
11–16.11	20 (18.5)
**Gender**	
Female	39 (36.1)
Male	69 (63.8)

**Table 2:** Intra-rater test-retest reliability results.

Item	Test (Mean ± SD)	Re-Test (Mean ± SD)	Interclass Correlation	Confidence Interval 95%	
				Lower Limit	Upper Limit
Band 1 (3–6.11 years)					
1	19.0 ± 7.16	17.4 ± 6.44	0.823	0.716	0.891
2	72.2 ± 35.6	68.7 ± 35.50	0.975	0.959	0.984
3	6.7 ± 4.86	6.4 ± 4.62	0.934	0.895	0.959
4	4.4 ± 3.04	5.5 ± 3.14	0.969	0.950	0.981
5	3.0 ± 1.94	4.1 ± 2.05	0.888	0.821	0.930
6	6.4 ± 6.5	6 ± 6.85	0.897	0.833	0.936
7	5.62 ± 4.58	5.6 ± 4.67	0.943	0.908	0.964
8	3.7 ± 1.56	3.9 ± 1.40	0.899	0.838	0.937
Band 2 (7–10.11 years)					
1	40.9 ± 11.27	37.2 ± 11	0.839	0.555	0.942
2	47.1 ± 18.82	46.4 ± 20.66	0.911	0.753	0.968
3	9.1 ± 12.12	4.2 ± 2.77	0.111	−1.456	0.678
4	5.0 ± 2.94	5.7 ± 2.21	0.904	0.734	0.965
5	3.9 ± 2.40	5.1 ± 2.19	0.908	0.746	0.967
6	3.6 ± 3.8	4.5 ± 3.26	0.039	−1.652	0.652
7	8.1 ± 4.87	8.1 ± 5.39	0.940	0.827	0.979
8	3.7 ± 1.49	4.1 ± 0.86	0.870	0.641	0.953
Band 3 (11–16.11 years)					
1	30.9 ± 5.1	27.7 ± 4.96	0.826	0.561	0.931
2	61.8 ± 29.70	50.4 ± 13.77	0.517	−0.221	0.809
3	1.5 ± 2.31	0.6 ± 1.05	0.233	−0.937	0.697
4	7.9 ± 2.1	8.3 ± 1.87	0.871	0.674	0.948
5	5.5 ± 1.99	5.1 ± 2.10	0.636	0.080	0.856
6	13.6 ± 10.12	17.0 ± 10.89	0.586	−0.047	0.836
7	11.2 ± 5.06	12.3 ± 4.76	0.887	0.714	0.955
8	4.6 ± 0.98	4.7 ± 0.76	0.140	−1.154	0.658

**Table 3:** Inter-operator test-retest reliability results.

Item	Rater 1 (Mean ± SD)	Rater 1 (Mean ± SD)	Interclass Correlation	Confidence Interval 95%	
				Lower Limit	Upper Limit
Band 1 (3–6.11 years)					
1	18.9 ± 5.99	19.05 ± 6.1	0.996	0.976	0.999
2	59.1 ± 9.494	59.3 ± 9.30	0.999	0.992	1.000
3	3.9 ± 4.059	3.9 ± 4.06	1.00		
4	5.6 ± 3.259	5.9 ± 3.29	0.986	0.921	0.998
5	4.3 ± 1.704	4.3 ± 1.70	1.00		
6	11.6 ± 10.17	11.3 ± 10.11	0.998	0.991	1.000
7	10.0 ± 5.598	9.7 ± 5.53	0.995	0.973	0.999
8	4.4 ± 1.134	4.1 ± 1.46	0.964	0.791	0.994
Band 2 (7–10.11 years)					
1	48.9 ± 10.2	48.7 ± 10.38	0.998	0.887	0.999
2	43.0 ± 25.24	43.0 ± 26.23	1.00	0.985	1.000
3	3.0 ± 1.73	2.7 ± 1.16	0.960	−0.560	
4	6.0 ± 3.61	6.0 ± 3.67	1.00		0.998
5	6.3 ± 2.08	7.0 ± 2.00	0.980	0.204	
6	2.5 ± 1.00	2.7 ± 1.06	0.902	−2.812	1.000
7	6.3 ± 7.51	6.3 ± 7.51	1.00		0.999
8	4.5 ± 1.73	4.2 ± 2.31	0.999	0.204	1.00
Band 3 (11–16.11 years)					
1	30.9 ± 5,1	31 ± 5.08	0.998	0.993	0.999
2	61.8 ± 29.70	61.3 ± 29.47	1.000	0.999	1.000
3	1.5 ± 2.31	1.4 ± 2.06	0.995	0.987	0.998
4	7.9 ± 2.12	7.5 ± 2.35	0.943	0.856	0.978
5	5.5 ± 1.99	5.5 ± 2.01	0.980	0.949	0.992
6	13.6 ± 10.12	13.0 ± 10.13	0.981	0.951	0.992
7	11.2 ± 5.06	11.3 ± 4.98	0.998	0.994	0.999
8	4.6 ± 0.98	4.5 ± 1	0.972	0.855	1.00

**Table 4:** Standard scores for band 1 (3–6.11 years old).

	Manual Dexterity				Aiming and Grasping		Balance			
Score	Item 1—P Hand	Item 1–NP Hand	Item 2	Item 3 Cat	Item 4 Cat	Item 5 Cat	Item 6—P Leg	Item 6—NP Leg	Item 7 Cat	Item 8 Cat
19	≤9	≤9	≤16.9	0	≥10	7	18<>30	20.7<>26.9	15–14	5
18		9.3–10.9	17–37.2	1	9	6	15<>18	15<>19.9		
17	9.1–10.4	11–13	37.3–44.3	2	8	5	12<>13.9	13<>15	13–11	
16	10.5–12	13.1–14.2	44.4–48.4				10<>11.9	10<>11.8	10	
15	12.1–13.6	14.3–14.9	48.5–51.9	3	7	4	10	9.3<>10	9	
14		15	52–54	4	6		7<>10	6<>8.6	8	
13	13.7–15	15.1–16	54.1–55				6<>7	5<>6	7	
12	15.1–16	16.1–17.9	55.1–57.4	5	5		5<>6	4<>5	6	
11		18<>19	57.5–60			3	5	4	5	4
10	16.1–18.9	19.1<>19.9	60.1–62.5	6	4		4<>5	3<>4	4	
9		20<>20.9	62.6–65.1				4	3		
8	19	21<>21.9	65.2–70		3		3<>4		3	
7	19.1–20.4	22<>22.9	70.1–77.3	7		2	3	2<>2.4		3
6		23<>23.7	77.4–85.4	8–9	2		3	2		
5	20.5–22	23.8–25	85.5–90	10			2–2.9		2	
4	22.1–23.9	25.1–25.9	90.1–101.4	11	1	1	2	1–1.6		2
3		26–28.9	101.5–112.4	12			1.9–2	1	1	
2	24–24.9	29	112.5–120		0	0	1–1.2			1
1	25–30	29.1–31.9	120.1–150.4	13–16			0.8–1	0.9–1	0	0
0	≥30	32–40.6	150.5–166.7	≥17			0<>0.1	0<>0.2		

**Table 5:** Standard scores for band 2 (7–10.11 years old).

	Manual Dexterity				Aiming and Grasping		Balance				
Score	Item 1—P Hand	Item 1—NP Hand	Item 2	Item 3 Cat	Item 4 Cat	Item 5 Cat	Item 6—P Leg	Item 6—NP Leg	Item 7 Cat	Item 8—P Leg Cat	Item 8—NP Leg
19	≤28	≤26	≤20	0	>10	≥8	≥7.6	≥9.6	≥15	5	5
18		26.1–26.9	20.8–28.9	1	9		6.2–6	9.5–6.1			
17	28.1–28.7	27–28.4	29–31.4	2		7	6–5.2	6–4			
16	28.8–29	28.5–32.6	31.5–32.6		8	6	5.2–5	3.9–3.5	14		
15	29.1–30.5	32.7–35	32.7–34		7	5	4.9–4.1	3.4–3	12–13		
14	31–32	35.1–36.2	34.1–35.8	2	6		4–3	3	11		
13	32.1–32.3	36.3–38.3	35.9–37.3	3–4	5	4	2.9–2.3		8–10		
12	32.4–33.2	38.4–39	37.4–38.2	5			2.4–2.9		7		
11	33.3–34.1	39	38.3–39.2				3			4	
10	34.2–35	39	39.3–41	6						3	
9	35.1–37.7	39.1–41.9	41.1–44.6			3	2		6		4
8	37.7–39.6	42	44.7–51.4		4				5	2	3
7	39.7–40.7	42.1–47.6	51.5–53					2.9–2.4			
6	40.8–41.6	47.7–51.8	53.1–55.4	7	3	2		2.3–2.1	4		
5	41.7–42.5	51.9–55	55.5–57.5	8	2		1.9–1.6	2–1.6	3		2
4	42.6–43.9	55.1–57.4	57.6–58.8	9	1	1	1.5–1.1	1.2–1.1	2		
3	44–46.2	57.5–60.7	58.9–63	10–20			1	1			
2	46.1–49.6	60.8–70.2	63.1–76	21–40	0	0	0.9–0.1			1	1
1	49.7–51.8	70.3–81.7	76.1–97.6	>40			0				

**Table 6:** Standard scores for band 3 (11–16.11 years old).

	Manual Dexterity				Aiming and Grasping			Balance			
Score	Item 1—P Hand	Item 1—NP Hand	Item 2	Item 3	Item 4—P Hand	Item 4—NP Hand	Item 5	Item 6	Item 7	Item 8—P Leg	Item 8—NP Leg
19	≤21–21.2	≤24	28–28.5	0	10	10	≥9	18.1–18.9	15	5	5
18	21.3–24.1	24.3–26.1	28.6–35.4				8	19–10			
17	24.2–25.3	26.2–27	35.5–39.3				7	21.7–29.9			
16	25.4–25.9	27.1–27.2	39.4–41.6					30			
15	26	27.3–27.9	41.7–44.3					30			
14	26.1–26.4	28	44.4–45.4				6	17.7			
13	26.5–27.5		45.5–45.9		9	9		17.6–15.8			
12	27.6–27.9	28.1–29.2	46		8			15.7–10.1			
11	28	29.3–31.5	46–47.4				5	10–11.9			
10		31.6–32.5	47.5–50.5					12–9.7	14		
9	28.1–28.6	32.6–32.9	50.6–52.6		7	8	4	9.6–8.4	11–13		
8	28.7–28.9	33	52.7–55.4	1				8.5–7.1	10		
7	29–31	33.1–33.7	55.5–58.9		6			7–4.5	9		
6	31.1–32	33.8–34	59–64.9			7		4.6–5.3	8		4
5	32.1–33.5	34–34.8	69.1–71.5	2		6		5.4–3.2	7–6		
4	33.6–36.9	34.9–36.9	71.6–84.2	3	5	5		3.2–2	5–4	4	3
3	37	37–38	84.3–109.6	4		4		2.1–2.9	3–2		
2	37.1–38.2	38.1–39.3	109.7–122	5			3	3	1	3–2	2
1	≥38.3	≥39.4	122.1–138.1	≥6		3				1	

**Table 7:** Percentiles.

Percentiles	Manual Dexterity	Aiming and Grasping	Balance
100°	55	38	57
75°	37	29	43
50°	26	19	34
25°	20	13	24
5°	11	4	10

## Data Availability

The complete dataset could not be published online due to the data privacy section of the informed consent approved by the local university ethical committee. Still, the aggregated data and materials may be available upon reasonable request from the corresponding author.
